# Diabetes, gall stone disease, and pancreatic cancer.

**DOI:** 10.1038/bjc.1986.185

**Published:** 1986-08

**Authors:** S. Norell, A. Ahlbom, R. Erwald, G. Jacobson, I. Lindberg-Navier, R. Olin, K. L. Wiechel


					
Br. J. Cancer (1986), 54, 377-378

Letter to the Editor

Diabetes, gall stone disease, and pancreatic cancer

Sir - Previous studies have indicated that there is
an association between diabetes and pancreatic
cancer. However, it is not clear to what extent this
could be explained by diabetic manifestations from
an early undiagnosed pancreatic tumour (Kessler,
1970; Wynder et al., 1973; Ragozzino et al., 1982;
Manousos et al., 1981). An association between gall
stone disease and pancreatic cancer has also been
suggested, but the findings have been somewhat
contradictory (Wynder et al., 1973; Manousos et
al., 1981; Haines et al., 1982). In a recent case-
control study we examined the association of
pancreatic cancer with previous diabetes and gall
stone disease.

All subjects aged 40-79 with a pancreatic cancer
diagnosed at three Swedish hospitals during the
study period (1982-4) were included as cases. Two
series of controls were used. Hospital controls were
selected as a stratified (age, sex) sample of subjects
aged 40-79 with newly-diagnosed inguinal hernia in
the same hospitals during the study period.
Population controls were selected from population
registers covering the catchment areas of the three
hospitals. Population controls were matched to the
cases by age, sex, and parish. More details on the
selection of subjects are given elsewhere (Norell et
al., 1986).

Each case received a questionnaire after
preliminary diagnosis, but only subjects whose
diagnoses were later confirmed were included in the

analysis. As soon as a case was found, a
questionnaire was mailed to the corresponding
population control. Hospital controls received a
questionnaire after clinical diagnoses. Whenever
necessary, subjects were contacted by telephone by
an interviewer to complete specific items in the
questionnaire. The numbers of eligible subjects (and
subjects who filled in the questionnaire) were: 120
(99) cases, 179 (163) hospital controls, and 162
(138) population controls. The Mantel-Haenszel
procedure was used for estimation of the relative
risk, accompanied by 90% test-based confidence
limits based on the Mantel-Haenszel test (Breslow
& Day, 1980).

The relative risk of pancreatic cancer for subjects
who reported that they had diabetes and gall stone
disease, respectively, are shown in the Table. To
exclude recent illness which could be an early
manifestation of cancer of the pancreas, we
reanalysed our data after exclusion of subjects who
reported that they had diabetes or gall stone disease
only during the last 5 years. However, there was
still an association with diabetes as well as with gall
stone disease, although the number of subjects with
diabetes was quite small. Due to the fact that some
subjects were severely ill, it was accepted that the
questionnaire was filled in by spouses for 16 cases,
2 hospital controls, and one population control.
When the data were reanalysed after the exclusion
of these subjects, the relative risks associated with

Table: Relative risk of pancreatic cancer associated with certain characteristics (together
with 90% confidence limits), estimated from comparisons with hospital controls and
population controls.

Hospital controls:      Population controls:
Cases:

Exposed     Exposed  Relative risk  Exposed   Relative risk

Exposure           number      number (90% conf lim)   number (90% conf lim)
Diabetes                    20           3   19.7 (7.8-50.1)    11    3.3 (1.7-6.4)
Gall stone disease          26         27     1.7 (0.9-3.0)     19    2.7 (1.5-4.7)
Diabetes

5+ years ago               4          2     6.9 (1.7-28.9)     3    2.4 (0.6-9.7)
Gall stone disease

5+ years ago              18         26     1.2 (0.6-2.3)     13    2.9 (1.5-5.6)
Biol relative with

diabetes                  26          17    2.7 (1.4-5.2)     36     1.0 (0.6-1.7)
Biol relative with

gall stone disease        40         46     1.4 (0.9-2.4)     51     1.3 (0.8-2.1)

378   S. NORELL et al.

diabetes increased somewhat while those associated
with gall stone disease were virtually unchanged.
There was also an association between pancreatic
cancer and reported diabetes and gall stone disease
in biological relatives. Our findings suggest that the
risk of pancreatic cancer is increased in subjects
with diabetes and gall stone disease, perhaps
because these diseases have some common
aetiologic factor with cancer of the pancreas.

Yours etc.

S. Norell and A. Ahlbom
Department of Epidemiology,
The National Institute of Environmental Medicine,

Stockholm.
R. Erwald
Department of Surgery,
Danderyd University Hospital,

Danderyd.

G. Jacobson
Department of Surgery,
Uppsala University Hospital,

Uppsala.
I. Lindberg-Navier
Department of Social Medicine,
Huddinge University Hospital,

Huddinge.

R. Olin
Preventive Unit for Occupational Medicine,

Royal Institute of Technology,

Stockholm.
K.-L. Wiechel
Department of Hepatobiliary and

Pancreatic Diseases,
S6dersjukhuset, Stockholm,

Sweden.

This work was supported by the Swedish Medical
Research Council (project No. 6616).

References

BRESLOW, N.E. & DAY, N.E. (1980). Statistical methods in

cancer research. Vol. 1: The analysis of case-control
studies. IARC Scientific Publications, No. 32; Lyon.

HAINES, A.P., MOSS, A.R., WHITTEMORE, A. & QUIVEY,

J. (1982). A case-control study of pancreatic
carcinoma. J. Cancer Res., Clin. Oncol., 103, 93.

KESSLER, J.J. (1970). Cancer mortality among diabetics.

J. Natl Cancer Inst., 44, 673.

MANOUSOS, O., TRICHOPOULOS, D., KOUTSELINIS, A.,

PAPADIMITRIOU, C., POLYCHRONOPOULOU, A. &
ZAVITSANOS, X. (1981). Epidemiologic characteristics
and trace elements in pancreatic cancer in Greece.
Cancer Detect. Prev., 4, 439.

NORELL, S., AHLBOM, A., ERWALD, R. & 5 others. (1986).

Diet and pancreatic cancer, a case-control study. Am.
J. Epidemiol. (in press).

RAGOZZINO, M., MELTON, L.J., CHU, C.-P. & PALUMBO,

P.J. (1982). Subsequent cancer risk in the incidence
cohort of Rochester, Minnesota, residents with
diabetes mellitus. J. Chron. Dis., 35, 13.

WYNDER, E.L., MABUCHI, K., MARUCHI, N. &

FORTNER, J.G. (1973). A case-control study of cancer
of the pancreas. Cancer, 31, 641.

				


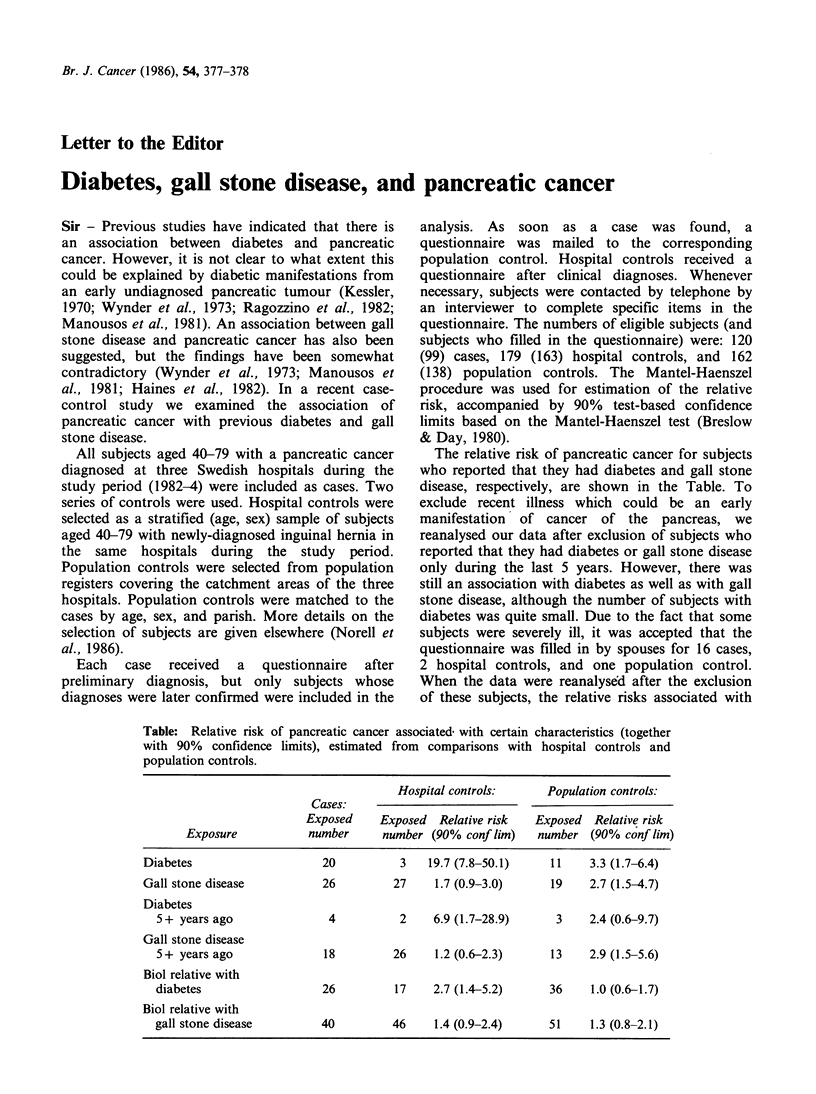

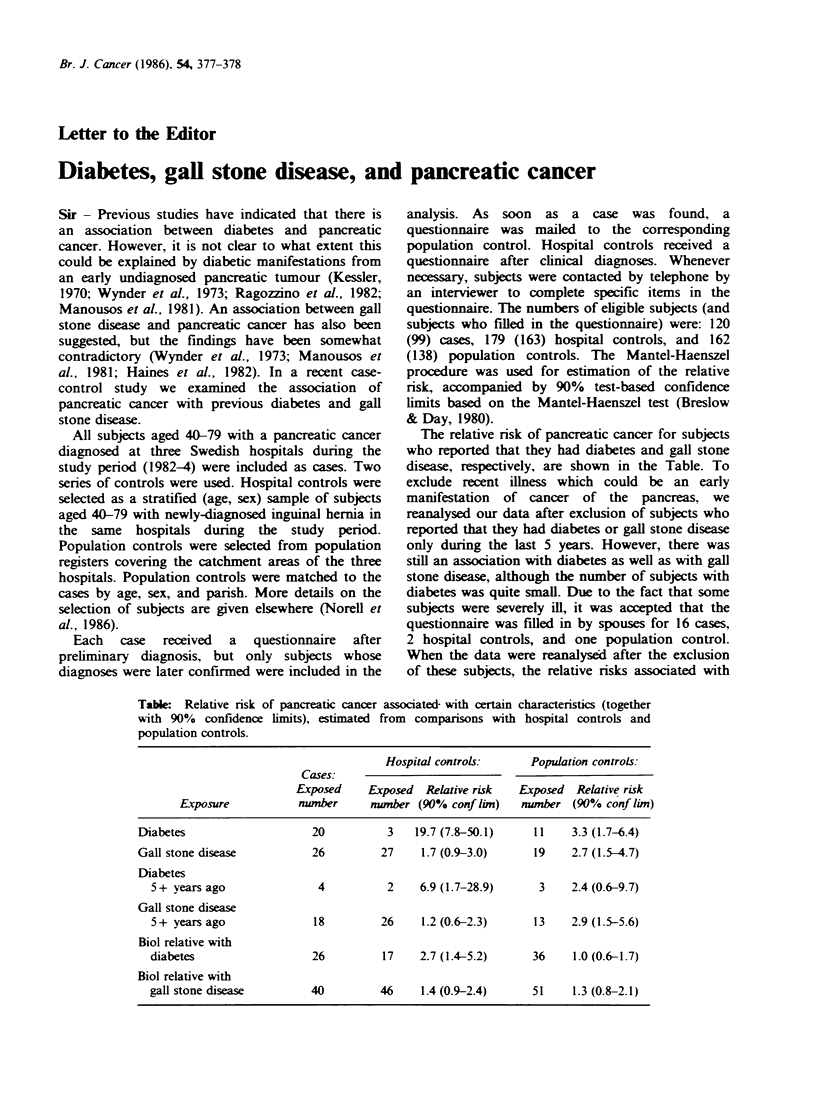

